# A Radar Echo Simulator for the Synthesis of Randomized Training Data Sets in the Context of AI-Based Applications

**DOI:** 10.3390/s24030836

**Published:** 2024-01-27

**Authors:** Jonas Schorlemer, Jochen Altholz, Jan Barowski, Christoph Baer, Ilona Rolfes, Christian Schulz

**Affiliations:** 1 Institute of Microwave Systems (HFS), Ruhr University Bochum, Universitätsstraße 150, 44801 Bochum, Germany; 2 Institute of Electronic Circuits (EST), Ruhr University Bochum, Universitätsstraße 150, 44801 Bochum, Germany

**Keywords:** computational EM, SAR, ground-penetrating radar (GPR), landmine detection, full-wave simulation

## Abstract

Supervised machine learning algorithms usually require huge labeled data sets to produce sufficiently good results. For many applications, these data sets are still not available today, and the reasons for this can be manifold. As a solution, the missing training data can be generated by fast simulators. This procedure is well studied and allows filling possible gaps in the training data, which can further improve the results of a machine learning model. For this reason, this article deals with the development of a two-dimensional electromagnetic field simulator for modeling the response of a radar sensor in an imaging system based on the synthetic aperture radar principle. The creation of completely random scenes is essential to achieve data sets with large variance. Therefore, special emphasis is placed on the development of methods that allow creating random objects, which can then be assembled into an entire scene. In the context of this contribution, we focus on humanitarian demining with regard to improvised explosive devices using a ground-penetrating radar system. This is an area where the use of trained classifiers is of great importance, but in practice, there are little to no labeled datasets for the training process. The simulation results show good agreement with the measurement results obtained in a previous contribution, demonstrating the possibility of enhancing sparse training data sets with synthetic data.

## 1. Introduction

In recent times, supervised learning algorithms such as deep neural networks (DNNs) and convolutional neural networks (CNNs) have proven to be quite powerful tools with regard to target classification tasks, as demonstrated in [1,2,3]. However, the huge potential of supervised learning approaches comes with the requirement of a much more complicated training process. In general, such algorithms require huge labeled data sets, with the number of data points scaling with the task to be solved. For instance, it was shown in the context of a classification problem in [4] that the amount of training samples for a nearly optimal solution should be from approximately 30·d·(d+1) to 60·d·(d+1). Here, *d* denotes the amount of nodes in the input layer. When evaluating the amount of necessary samples based on the found formulas, it turns out that generating sufficient training data sets for complex problems becomes a tedious task. While this is a time-consuming but manageable task in many applications, there are measurement environments that do not allow collecting huge data sets or even no training data at all. The reasons for this could be manifold. A typical problem is that the true state of the system is quite difficult to determine in advance, preventing adding labels to the data set. Furthermore, the number of possible states is often too large to generate meaningful training data, covering all possible states of the system. For those cases, the available training data sets are usually too small.

As an exemplary application scenario, we focus in this paper on the field of humanitarian demining, since this application usually lacks comprehensive labeled data sets. This is caused by the complexity and uncertainty in labeling a measurement without interacting with the environment. While this is a general problem in the field of demining, it is often possible to simplify the problem if only a certain type of landmine is expected in the measurement environment. As this is often the case, there are multiple publications that propose systems and classifiers which aim to detect professionally built landmines as in [5]. However, a much more complicated task is the detection of improvised explosive devices (IEDs). In contrast to professionally built landmines, IEDs are built out of everyday objects and are often used by paramilitary groups, such as those in Colombia, due to their comparatively simple production. This type of landmine is significantly harder to detect, as basically all IEDs are unique in their structure, and a sensor system responds differently to each realization of an IED. For this reason, the requirements for training data sets are significantly greater in comparison with data sets for professionally built landmines, as not only must the variation of the measurement environment be modeled but also the variation of the IED itself. Furthermore, the sensor system must be designed carefully to detect all features of buried IEDs.

Recent publications have suggested the use of ground-penetrating radar (GPR) for landmine detection. GPR systems provide a raw data matrix obtained from multiple measurements carried out by a moving platform. In principle, all platforms that allow spatial displacement of the sensor are suitable for carrying out the measurements. A common approach is the use of unmanned aerial vehicles (UAVs), such as in [6,7,8,9], as these do not interact with the earth surface during the measurement process. This approach is particularly advantageous in large, non-vegetated environments. However, in environments with heavy vegetation, the use of flying platforms is difficult to realise. The use of robots [10] and handheld sensors [11] is particularly important here. In the context of humanitarian demining, a landmine leads to specific spatial reflection signatures in the GPR raw data, as shown in [12,13,14]. A system that applies a similar measurement method is ground-penetrating synthetic aperture radar (GPSAR), which uses a more advanced signal processing procedure following a synthetic aperture radar (SAR) approach. Such systems as the one in [6,8,9] allow one to form high-resolution images of objects beneath an earth surface. However, this requires accurate modeling of the wave propagation, where it is mandatory to measure the soil material parameters in advance. The great advantage of radar-based systems is that they allow one to obtain information on buried objects without mechanically interacting with the measurement environment, which is of importance in humanitarian demining. Furthermore, they are especially of importance for detecting IEDs since they respond to changes in wave impedance, whereby non-metallic, irregularly shaped objects can also be detected. On the downside, radar approaches typically suffer from clutter in multi-target environments, which is inflicted by multiple reflections. As was shown in [15] by means of simulations, the amount of clutter in the received signal heavily depends of the orientation of the antenna. In general, it can be stated that a down-looking radar configuration provides a better resolution with an increased amount of clutter, while the resolution of a side-looking radar is typically smaller but provides a lower clutter level. Another disadvantage of radar-based systems is the fact that the penetration depth decreases considerably if the ground material has high losses, which is why extremely deeply buried objects are lost in the noise and therefore, cannot be detected. In order to minimize losses, radar sensors with a transmission signal in a relatively low frequency range up to around 5GHz are typically used.

Aside from radar-based systems, there are several other approaches to designing a sensing system for humanitarian demining. This primarily involves the use of magnetometers and metal detectors [11,16], which aim to detect the magnetic field generated by buried metal pieces in the presence of a time-varying electromagnetic field. Although this method is frequently used, it is often difficult to distinguish landmines from other metal objects, and thus the sole use of metal detectors leads to a high false alarm rate. Furthermore, IEDs can be manufactured with a rather small amount of metal pieces, which is why the use of metal detectors alone is not effective. Another approach to humanitarian demining is infrared sensors, which aim to track the heat signatures of landmines. It was shown in [17] that this procedure works well if the landmines are placed on top of the surface, but the detection accuracy decreases the deeper the mine is buried underground, as the thermal signatures are increasingly influenced by the surrounding material. Since all named sensors, including the radar approach, are prone to errors, it is a current research topic to design systems which rely on several complementary sensors. An example is the system in [18], which relies on a GPR approach combined with a magnetometer.

All mentioned sensors provide data that are difficult for the user to interpret, and a trained classifier is required. In this paper, we focus on the problem of generating training data for GPR and GPSAR systems, as these systems are the most promising ones for the detection of IEDs. Here, we investigate handheld devices, where the sensor is typically placed at a short distance from the earth surface in a down-looking configuration. This work builds on measurement campaigns carried out by our research group in the past. The processed measurement results recorded by our GPSAR system in an environment with buried IEDs can be found in [19].

To tackle the problem of missing training data, one possible solution is to manufacture artificial mines that behave as much as possible like real landmines. These artificial mines can then be buried in an environment that behaves like the intended measurement environment to collect realistic labeled measurements. An example can be found in [5], where true measurements were recorded with professionally manufactured landmines. Here, online dictionary learning was used for advanced feature extraction before feeding the preprocessed data to a support vector machine (SVM). It was shown that the proposed algorithm outperformed conventional deep learning methods while requiring significantly less training data. However, building data sets based on true measurements requires capturing all possible realizations of a landmine. As stated, this is possible for professionally manufactured landmines but rather challenging for IEDs due to the huge amount of possible realizations. Therefore, the procedure of collecting true measurements is not effective, as only a small amount of possible IEDs can be investigated. For instance, in [20], a total of 30 different IEDs were manufactured to obtain realistic training data. Afterward, an SVM trained with the measured training data reached an accuracy of up to 87.02%. While this is a promising result, classifiers for humanitarian demining must reach an accuracy of almost 100%, especially if handheld devices are used. Another approach to get around the problem of missing data is to handle landmines as anomalies, as shown in [21]. Here, an autoencoder was trained purely on measurement data that did not contain a landmine, reaching an accuracy of up to 93%.

A well-studied solution to dealing with missing training data is to use synthetic data generated by an appropriate simulator which is designed to mimic the true measurement environment. This approach provides the advantage that the user has complete control over the simulation environment, where automatic labeling of the data is an easy task. However, it was shown in [22] that training a classifier purely based on simulated data sets leads to bad results. The reason for this can be found in the circumstance of it being nearly impossible to model a realistic scenario including the measurement system itself. On the other hand, it was also shown that enhancing sparse data sets with simulated data increases the performance of the neural network, outlining the possibility of filling gaps with simulated data sets. A related approach is transfer learning, where a machine learning model is pretrained with an available data set that does not necessarily belong to the true measurement environment. In practice, this shows that data-driven machine learning models are able to transfer the knowledge from the pretrained model to the actual data, allowing one to use sparse training data sets. For instance, it was shown in [23] in the context of motion classification that it is beneficial to use training data generated by a sufficiently accurate simulator to pretrain the classifier. Here, the classification results improved by 9% using simulated data. In [24], it was shown that a CNN intended for target recognition could be trained with data generated by a commercial three-dimensional simulator, leading to faster convergence when trained with the real data set. Another example can be found in [25], where the CNN was pretrained with simulated SAR images, showing that the fine-tuned neural network outperformed a conventional CNN when the training data set was enhanced by simulated data.

The principle of using synthetic data is also a common approach in landmine detection systems. For this, there are multiple publications using the solver gprMax, such as [26,27,28,29], to simulate a fully three-dimensional GPR environment by applying a GPU-accelerated finite-difference time domain (FDTD) method. In [30], it was shown by means of a region-based convolutional neural network (R-CNN) that the classification accuracy of a GPR system can be increased if the true measurements are extended by simulated data. In [31], a CNN was trained exclusively with data obtained from gprMax while being tested on real measurements. It was shown that the classifier was capable of reaching a classification accuracy of 83% by only using synthetic data for training. Furthermore, it was shown that adding the true measurements of scans without landmines to the training data increased the detection accuracy up to 95%. Aside from these examples, there are also publications that explicitly deal with modeling IEDs in simulations. In [14], an R-CNN was trained with real and simulated data to detect the characteristic hyperbolic patterns of IEDs. Here, the simulated data consisted of simulations with gprMax as well as hand-drawn hyperbolas, while the accuracy was evaluated with the intersection over union (IoU) metric. It was shown that the trained R-CNN model reached an IoU of up to 96.68%. In [12], an IED was modeled with a camping cylinder to investigate the possibility of knowledge transfer from different sensing environments. Here, it was shown that the trained least squares support vector machine (LS-SVM) was capable of transferring the learned model to other measurement environments by only using a rather limited amount of new training data. In [13], a total of five different landmines were modeled. The IEDs were modeled, for instance, as plastic canisters filled with the explosive material. However, all publications concerned with IEDs have in common that the IEDs had to be modeled by hand, and thus only a few specific building types could be investigated. To the authors’ best knowledge, there are no publications until today that not only model a random measurement environment but also take care of the random building type of the IED itself. For this reason, this publication aims to develop a simulation framework that allows generating fully randomized training data sets by also applying a randomized mine model for IEDs.

While three-dimensional simulators like gprMax allow more accurate modeling of the problem, they suffer from a much slower computation time. Regarding two-dimensional simulation schemes, the simulator presented in [32] applies a two-dimensional FDTD method to generate the raw data. The measurement environment is modeled by a randomized earth surface and a randomly distributed soil permittivity while including bullets as false alarm targets. However, the authors of [26,27,28,29,32] dealt with professionally built landmines by calculating the time domain raw data matrix. In contrast, this contribution aims to model IEDs while applying an appropriate postprocessing of the raw data. Furthermore, the model presented in this publication includes statistically generated targets and a randomly generated mine model, resulting in a more complex simulation environment.

**Figure 1:**
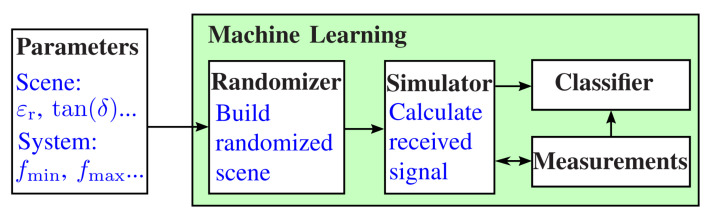
Procedure of the training process. The description parameters are passed to a randomizer, which generates the scene for the simulator. The simulator generates the training data, which are cross-validated with the real measurements and then used to train the classifier.

The simulation environment presented here refers to the system we proposed in [19]. This is a handheld M-sequence radar device that emits electromagnetic waves in the low-frequency range of up to 5GHz. In general, however, the underlying simulation approach is independent of the radar system and can also be applied to other frequency ranges. Therefore, the presented simulation approach allows modeling the received signals of future modern radar sensors in numerous applications. Figure 1 shows the basic principle of the proposed training process. The describing parameters of the environment, like the relative permittivity $\varepsilon_{r}$, and the parameters of the measurement system are passed to a randomizer. This generates a random scenario of the environment, which is used to calculate the received signal by means of a full-wave simulation. The simulation results are validated by measurements of the sensing environment to ensure that the simulated data match the measured data with sufficient accuracy. Afterward, the simulated data and the true measurements are used to train the classifier.

This manuscript is organized as follows. Section 2 gives insight into radar imaging using the backprojection algorithm, including the refraction compensation in a GPR system. In Section 3, we introduce the basic simulation concept, namely the FDFD method. Afterward, two approaches are presented in Section 4 which allow generating random objects by means of filtered noise. Furthermore, the generation of randomized IED models is presented in this chapter. Section 5 provides an overview of the implementation of the simulator, while Section 6 and Section 7 then show some selected simulation results for a simplified mine model and the IED models, respectively. Finally, Section 8 provides a conclusion of the obtained results.

## 2. Radar Imaging

In the following, we will give a brief introduction into the postprocessing procedure of the raw data obtained by the simulation. Here, we will start with the range-compressed raw data $\underline{s}\left(\tau \right)$ and focus on the radar imaging algorithm to obtain images from objects beneath the earth surface. For a more in depth discussion on the underlying signal model, we refer to [33].

Assuming that the wave propagates with the speed of light $c_{0}$, the target is at a constant distance *R* and the round-trip time is given by $\tau_{0}=\frac{2\cdot R}{c_{0}}$, the range-compressed signal can be described as follows:

(1) $$\underline{s}\left(\tau \right)=sinc(\frac{\Omega }{2}\left(\tau -\tau_{0}\right))\cdot \exp\left(-j2\pi f_{0}\tau \right)$$

where Ω denotes the bandwidth of the system, while $f_{0}$ is the center frequency of the emitted signal. By following this approach, one can achieve one-dimensional range information from a single measurement. However, to obtain two- or three-dimensional results, one has to carry out several measurements. For instance, in case of a synthetic aperture, a single radar sensor is moved above the target area, while multiple measurements are taken at different positions along the trajectory. Afterward, the image of the measurement environment is formed by applying a pulse compression in the range and cross-range directions. Assume that the sensor moves along an arbitrary trajectory and carries out *M* measurements, where ${\vec{r}}_{a,m}$ denotes the *m*th measurement. For a point scatterer at the position $\vec{r}$, the round-trip time can be found with $\tau_{m}=\frac{2∥{\vec{r}}_{a,m}-\vec{r}{∥}_{2}}{c_{0}}$. With the system parameters known a priori, the range-compressed signal can be found by substituting the round-trip time into Equation (1). After range compression, cross-range compression can be achieved by means of the backprojection algorithm, among others. For this, we assume that the target area can be approximated by a set of discrete point scatterers. Following this, the reflection at a discrete point in space can be found by adding up the corresponding values in the range-compressed signals after compensation of the phase term. By repeating this for all *M* measurements at a sufficiently large amount of points in space, one can form an image $\underline{I}\left(\vec{r}\right)$ of the measurement environment. For a single discrete point ${\vec{r}}_{t}$ in space, the formula is as follows:

(2) $$\underline{I}\left({\vec{r}}_{t}\right)=\frac{1}{M}\sum_{m=1}^{M}\underline{s}\left(\tau_{m}\right)\cdot \exp\left(j2\pi f_{0}\tau_{m}\right)$$

**Figure 2:**
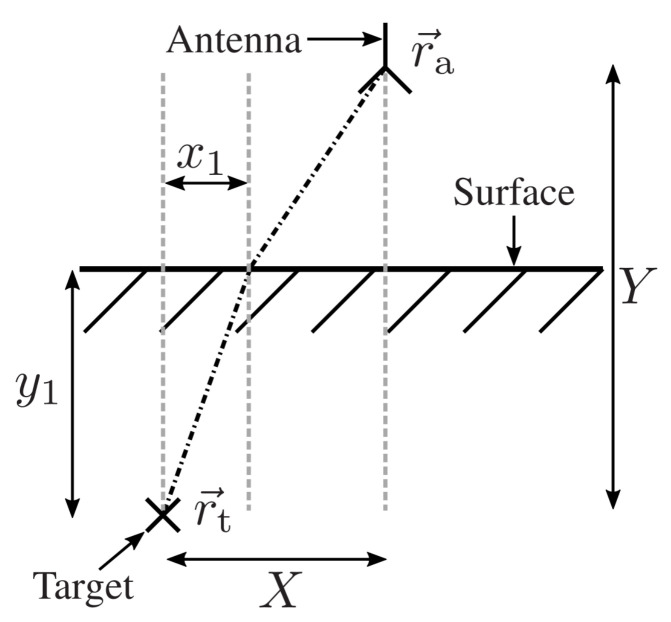
Refraction situation for a single antenna position with an idealized point target buried in soil material with a flat surface.

The found formula is a straightforward solution for the imaging problem. However, backprojection of the range-compressed signal relies on the modeling of the wave propagation and the according round-trip time $\tau_{m}$. In case of a GPR system, the surrounding medium is inhomogeneous, where the refraction effects at the earth surface and varying propagation speeds have to be taken into account. Therefore, provided that the soil permittivity is known a priori, the main problem is to find the refraction point on the earth surface. As was shown in [34], this can be achieved by evaluating Snell’s law. Figure 2 shows the measurement scenario of an antenna located at ${\vec{r}}_{a}$ over the soil material and the observation point ${\vec{r}}_{t}$ below the earth surface. We denote the overall horizontal distance between the antenna and the observation point with *X* and the horizontal distance between ${\vec{r}}_{t}$ and the refraction point with $x_{1}$. Similarly, *Y* denotes the vertical distance between the antenna and the observation point, and $y_{1}$ is the distance between ${\vec{r}}_{t}$ and the earth surface. In reference to Figure 2, the problem reduces to finding $x_{1}$. According to Snell’s law, and by applying some calculations, the following equation can be derived:

(3) $$\begin{array}{c} x_{1}^{4}\left(N^{2}-1\right)+2Xx_{1}^{3}\left(1-N^{2}\right)+x_{1}^{2}(N^{2}X^{2}+N^{2}y_{1}-X^{2}\\ \;-Y^{2}+2Yy_{1}-y_{1}^{2})-x_{1}\left(2Xy_{1}^{2}N^{2}\right)+N^{2}X^{2}y_{1}^{2}=0 \end{array}$$

Here, *N* is the ratio of the refraction index $n_{1}$ of the material above the earth surface and the refraction index $n_{2}$ of the soil material. In the literature, there are solutions to find the roots of such functions, and the theory ensures Equation (3) always provides four complex roots. However, in the case of the given GPR problem, the solution can be restricted to the positive real-valued root. After calculating $x_{1}$, the round trip time can be found as follows:

(4) $$\tau =\frac{\sqrt{{\left(X-x_{1}\right)}^{2}+{\left(Y-y_{1}\right)}^{2}}}{c_{0}}n_{1}+\frac{\sqrt{x_{1}^{2}+y_{1}^{2}}}{c_{0}}n_{2}$$

Finally, substituting Equation (4) in Equation (2) solves for the refraction problem, allowing the forming of images of objects below the earth surface.

## 3. Simulation Concept

In this section, we provide an introduction to the simulation of radar signals using the FDFD algorithm. For a more detailed discussion of the numerical method and the implementation, we refer to [35,36].

It can be shown that the received signal of the radar sensor is equal to the transfer function of the measurement environment, except for a constant factor. This is a generally valid relationship that is not limited to the field of GPR. Therefore, the problem is equivalent to calculating the field distribution at the receiver for a given excitation of the source. For this, one could rely on asymptotic simulation concepts like ray tracing or physical optics to calculate the received signal. However, the named methods are powerful approaches for free space applications but usually lack accuracy in more complex environments. Therefore, we rely on mesh-afflicted methods, which can be adopted to all simulation scenarios at the cost of much larger computational complexity. As it is known from theory, the description of electromagnetic phenomena in the time and frequency domains is completely equivalent. Therefore, the simulation domain can be chosen arbitrarily as long as the boundary conditions provide a unique solution. However, effects like losses and dispersion are usually introduced in the frequency domain, whereas modeling the scenario in the frequency domain is the more straightforward solution.

While time domain methods like the FDTD method aim to propagate an incoming wave along a discrete grid over time, frequency domain solvers produce a linear equation system, which must be solved independently for each frequency. Through this, the received signal of the radar sensor can be evaluated at discrete samples, where each sample is found by a separate simulation. Regarding full-wave solvers in the frequency domain, the finite element method (FEM) is state of the art. This can mainly be justified by the ability to adopt the underlying mesh to the simulation environment, resulting in a sparse equation system. However, generating a good variational grid is a complex task, especially when dealing with inhomogeneous materials like in this contribution. Therefore, we rely on the FDFD method, since the mesh can be generated comparatively quickly for an arbitrary material distribution. In terms of the investigated simulation environment, this is a significant advantage that simplifies the simulation considerably but at the price of a larger system of equations to solve.

The basis of the FDFD method is the approximation of the differential operator by a finite difference. We are using a central finite difference, which can be formulated as follows for a differentiable function f(x):

(5) $$\frac{\partial f(x)}{\partial x}\approx \frac{f\left(x+\frac{\Delta }{2}\right)-f\left(x-\frac{\Delta }{2}\right)}{\Delta x}$$

Here, Δx denotes the spacing between two function values used for the approximation. The result in Equation (5) is an important one, but it leads to the problem that the function values used for the approximation are only known at integer multiples of Δx, where the values at $x\pm \frac{\Delta x}{2}$ must be interpolated. However, from Maxwell’s equations it is known that $\vec{E}\propto \nabla \times \vec{H}$ and $\vec{H}\propto \nabla \times \vec{E}$, through which it becomes clear that $\vec{E}$ and $\vec{H}$ can be staggered in space with a spacing of $\frac{\Delta x}{2}$. In practice, this is solved by applying the Yee grid as was shown in [37]. Aside from the problem of staggered fields, this grid also assures that the divergence equations of the electromagnetic field are fulfilled automatically while allowing an efficient approximation of the curl equation.

To transfer the finite difference principle to the scattering problem in the GPR context, we evaluate the wave equation for the electric field $\vec{E}\left(\vec{r},\omega \right)$ in the frequency domain:

(6) $$\begin{array}{c} \nabla \times \mu^{-1}\nabla \times \vec{E}\left(\vec{r},\omega \right)+\omega^{2}\varepsilon \vec{E}\left(\vec{r},\omega \right)=0 \end{array}$$

Here, μ and ε denote the permeability and permittivity, respectively, while ω denotes the angular frequency. The equation describes the field distribution of the electric field in the absence of sources. However, this equation cannot be solved on a computer since we are dealing with a continuous spatial variable $\vec{r}$. To reduce this to a manageable problem, one can introduce the Yee mesh cells, whereas the curl operator can be described as a multiplication with the matrices $C_{H},C_{E}\in R^{N\times N}$. Here, $C_{H}$ is the finite difference approximation for the magnetic field on a discrete grid with *N* Yee cells. Accordingly, $C_{E}$ denotes the matrix for the electric field. Substituting the electric field in Equation (6) with its discrete equivalent and the curl operators with the according matrices leads to a fully three-dimensional solver for the problem setting. However, applying a three-dimensional simulation of the problem leads to high demands for memory and computational power. Therefore, we meet a two-dimensional approximation by ensuring $\frac{\partial }{\partial \;z}\vec{E}\left(\vec{r},\omega \right)=0$. Following this, the wave equation decouples in two independent modes, namely the E and H modes. In the further explanations of this section, we refer to the E mode with the field components $H_{x},H_{y}$, and $E_{z}$. The formulas for the H mode can be found in a similar way.

After introducing the material parameters $\mu_{f}$ and $\varepsilon_{f}$ of the free space, the discrete wave equation for the E mode is as follows:

(7) $$\begin{array}{c} \left(C_{H}\mu^{-1}C_{E}+\omega^{2}\varepsilon \right){\vec{E}}_{z},\mathrm{sc}=-\left(C_{H}\mu_{f}^{-1}C_{E}+\omega^{2}\varepsilon_{f}\right){\vec{E}}_{z},\mathrm{inc} \end{array}$$

For this solution, it was assumed that the field could be divided into the incoming field ${\vec{E}}_{z,\mathrm{inc}}$ and the scattered field ${\vec{E}}_{z,\mathrm{sc}}$, with ${\vec{E}}_{z}={\vec{E}}_{z,\mathrm{inc}}+{\vec{E}}_{z,\mathrm{sc}}$. Equation (7) finally allows calculating the electric field by introducing the source by means of the incoming field and solving the equation system afterward. However, attention must be placed on the boundary conditions, since the finite difference approach in Equation (5) naturally requires field components outside of the discretized space, leading to eventual reflections. An effective approach to avoiding such reflections is the perfectly matched Layer (PML). Here, an anisotropic lossy material is placed around the simulation domain that allows inducing losses without applying reflections to the incoming wave.

Finally, after implementing the finite difference approximation of the wave equation including the PML, the scattered field can be calculated by using a total-field scattered-field (TFSF) implementation of the source. Using this implementation ensures that the emitted wave can only propagate in the TF region, while the SF region only contains the scattered wave components. Therefore, one can separate those two field components at the receiver and find the received signal by calculating the ratio $\frac{{\vec{E}}_{z},\mathrm{sc}}{{\vec{E}}_{z},\mathrm{inc}}$.

## 4. Randomized Simulation Scenes

As already mentioned in Section 1, it is of great importance that the generated data contain large variance, meaning that the generated scenes are as diverse as possible. To ensure this, a mechanism is required that allows scenes to be generated randomly. Therefore, we will introduce two related methods in this section which allow generating random objects. Here, we trace the problem back to white noise, which can be generated effortlessly nowadays.

### 4.1. Randomizing Contours

Formally, we consider a stochastic process where Ω is the set of possible outcomes, while ω∈Ω denotes one specific outcome. We limit ourselves to discrete stochastic processes and define $X\left(\omega ,n\right)={\left[X_{1},\ldots ..,X_{N}\right]}^{T}$ as a mapping of the outcome ω on a set of *N* random variables. For the sake of simplicity, we drop the dependency on ω and simply denote the stochastic process with X(n). Let X(n) be normally distributed for all n∈[1,N] with a mean value of EX(n)=0, a variance $EX{\left(n\right)}^{2}=\sigma^{2}$, and the covariance $Cov(X_{n},X_{m})=0\;\forall n\neq m$. In this case, we call X(n) white noise, and it can be shown that X(n) has a constant power density spectrum.

We now aim to generate a random curve by applying an appropriate filter. Filtering can either be carried out by evaluating a convolution in the time domain or a multiplication in the frequency domain. Here, we use the frequency domain solution because the calculation is usually faster due to efficient implementation of the fast Fourier transform (FFT).

As already stated, white noise provides a constant power density spectrum, while the absolute majority of all Fourier coefficients is not equal to zero. This leads to curves with rather high slopes, as can be shown for a continuous function x(t). Assume that there is a pair $x\left(t\right)⊶\underline{X}\left(f\right)$. One can show that the following expression holds for a symmetrically defined Fourier transform:

(8) $$\left|\frac{dx(t)}{dt}\right|\leq \sqrt{2\pi }\int_{-\infty }^{\infty }\left|\underline{X}\left(f\right)\cdot f\right|df$$

Therefore, there is an upper bound for the slope which can be expressed by the spectrum. Following this, for a fixed spectrum $\underline{X}\left(f\right)$, one can decrease the upper bound by applying a low-pass filter with the impulse response $\underline{H}\left(f\right)$, leading to a continuous curve with a decreased slope. For this reason, we define the discrete filter transfer function $\underline{H}\left(f_{m}\right)$ as a Gaussian filter:

(9) $$\underline{H}\left(f_{m}\right)=\frac{1}{\sqrt{2\pi \sigma^{2}}}\cdot \exp\left(-\frac{f_{m}^{2}}{2\sigma^{2}}\right)$$

with $\sigma^{2}$ denoting the variance of the filter and $m\in [-\frac{M}{2},\frac{M}{2}-1]$ for an FFT length of *M*. Let $\underline{X}\left(f_{m}\right)=F\left\{X\left(n\right)\right\}$ be the frequency domain representation of a stochastic process. Then, the filtered signal $\tilde{X}\left(n\right)$ in the time domain can be found as follows:

(10) $$\tilde{X}\left(n\right)=F^{-1}\left\{\underline{H}\left(f_{m}\right)\cdot \underline{X}\left(f_{m}\right)\right\}\left(n\right)$$

Since the generated noise is real-valued, the spectrum is symmetric, and due to the symmetry of the filter, the resulting signal $\tilde{X}\left(n\right)$ is real-valued as well. In fact, applying the filter in Equation (9) sets most Fourier coefficients to zero or at least close to zero, except for only a few coefficients close to the origin of the spectrum. Subsequently, $\tilde{X}\left(n\right)$ can be mapped to a certain co-domain $\left[x_{\min},x_{\max}\right]$ by applying the following transformation:

(11) $$\hat{X}\left(n\right)=\frac{\tilde{X}\left(n\right)-\min\left\{\tilde{X}\right\}}{\max\left\{\tilde{X}\right\}-\min\left\{\tilde{X}\right\}}\cdot \left(x_{\max}-x_{\min}\right)+x_{\min}$$

**Figure 3:**
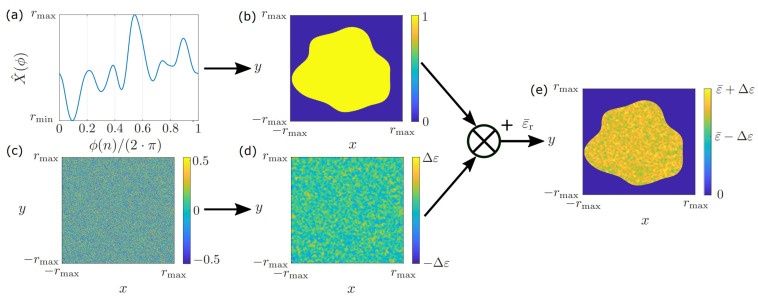
Generation of a random object. (**a**) Randomized curve which can be interpreted as the radius, depending on the angle. (**b**) Interpolation of the curve on a two-dimensional grid using a polar coordinate system. (**c**) Two-dimensional noise. (**d**) Filtered noise using a Gaussian filter. (**e**) Final object.

Based on the randomized curve $\hat{X}\left(n\right)$, one can build random objects by transferring the concept to the desired form of the object. For instance, objects from the field of GPR that can be modeled by line elements, such as branches, can easily be modeled by the presented concept. However, the presented method is also a powerful tool for generating random two-dimensional contours, as can be seen in Figure 3. Here, Figure 3a shows a random curve generated by filtered noise, where the curve can be interpreted as the radius $r\in [r_{\min},r_{\max}]$ depending on the angle ϕ(n)∈[0,2π]. Afterward, the curve is fitted on a two-dimensional grid as shown in Figure 3b, with the contour given by

(12) $$C\left(n\right)=\hat{X}\left(n\right)\cdot {\left(\cos\left(\phi \left(n\right)\right),\sin\left(\phi \left(n\right)\right)\right)}^{T}$$

Subsequently, the object is filled with ones everywhere inside the contour and zeros elsewhere, resulting in a binary mask of the object. Since most real-world objects that can be found in GPR systems are strongly inhomogeneous, randomized objects cannot be modeled by setting the relative permittivity to a constant value. One can account for this fact by generating two-dimensional noise like in Figure 3c and applying a two-dimensional Gaussian filter, which results in the distribution shown in Figure 3d. Here, the values inside the matrix are scaled to an interval [−Δε,Δε] by Equation (11), which accounts for the dispersion of the permittivity within a given interval. Subsequently, the final object is formed by multiplying the filtered noise in Figure 3d by the binary mask in Figure 3b. Thereby, the filtered noise is set to zero everywhere outside the object. Furthermore, we add a mean relative permittivity value ${\bar{\varepsilon }}_{r}$, while we obtain the final object in Figure 3e, which contains a random contour and a randomized relative permittivity distribution.

The arising problem in designing randomized objects is to select the parameters in the presented procedure so that the generated objects are close to the real targets. The two important parameters that have to be chosen in the proposed algorithm are the standard deviation σ of the applied filter transfer function as well as the permittivity of the objects. In practice, choosing these parameters requires a detailed analysis of the measurement environment. Here, material characterization is of central importance, as parameters such as the relative permittivity have a great influence on the simulation results and must be known a priori for as many objects as possible. Regarding the standard deviation of the filter, there are no fixed exact values, as these values strongly depend on the geometry of the intended object. However, regarding two-dimensional objects like in Figure 3, for example, the rule of thumb is to choose σ to be relatively small, with σ<50Hz if the object is close to a sphere. The larger the standard deviation, the larger the curvature of the resulting filtered curve will be, and the more the resulting two-dimensional object will differ from a sphere. As with the relative permittivity, the standard deviation is ideally selected on the basis of an investigation of the possible geometries in the environment. However, following the approach of transfer learning in the introduction, it is not of central importance to exactly recreate the overall structure of all possible objects exactly but to present the classifier training data with a certain variance, since machine learning models are typically able to transfer knowledge from one problem to another. For instance, the R-CNN in [14] was trained to classify the hyperbolic pattern of IEDs while being partially trained with hand-drawn hyperbolas. Furthermore, in [12], it was shown that a classifier trained for detection in a certain environment can be transferred to another environment with only a few new training samples.

### 4.2. Including a Priori Knwoledge

The presented method in Section 4.1 is well suited for generating objects with completely random contours, as shown in Figure 3. However, many real-world objects contain sharp edges or can be modeled as a variation of a specific geometrical shape. For instance, IEDs are often build from bottles or plastic containers, which usually do not offer a completely random shape. Therefore, it is of great importance to consider a priori knowledge of the shape in the randomized generation process. However, modeling such objects with the presented method in Section 4.1 is a tedious task, since it requires manipulating the obtained random curve. A more suitable method for including a priori knowledge is to model the object with a continuous deformation of a reference object. In the following, we will describe the procedure for the case of a continuous variable since it simplifies the discussion. We will then transfer the method to the discrete domain.

Let t∈[0,1] be a continuous variable and $\vec{F}\left(t\right)$ be a closed continuous curve in $R^{2}$, which we will call the reference. Furthermore, let ϕ(t)∈[0,2π] be a continuous curve with ϕ(0)=ϕ(1). We now want to create a new curve based on a continuous deformation of the reference $\vec{F}\left(t\right)$. Therefore, we define the following curve using polar coordinates:

(13) $$\vec{D}\left(t\right)={\left(\cos\left(\phi \left(t\right)\right),\sin\left(\phi \left(t\right)\right)\right)}^{T}$$

Using this result, a continuous deformation ${\vec{F}}^{\prime }\left(t\right)$ of $\vec{F}\left(t\right)$ can be expressed as a weighted sum of $\vec{F}\left(t\right)$ and $\vec{D}\left(t\right)$:

(14) $${\vec{F}}^{\prime }\left(t\right)=\vec{F}\left(t\right)+\alpha \left(t\right)\cdot \vec{D}\left(t\right)$$

**Figure 4:**
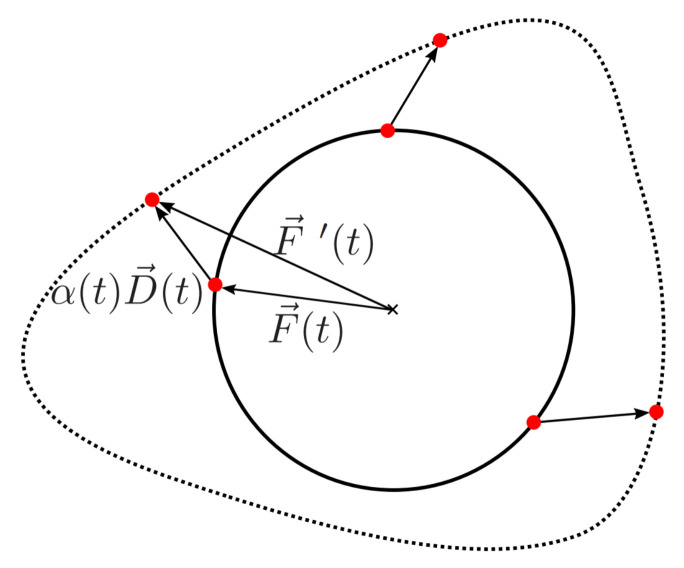
Continuous deformation of a reference object.

Here, α(t) is a weighting factor, which can be chosen arbitrarily. However, we demand that ${\vec{F}}^{\prime }\left(t\right)$ should be closed and continuous, which forces α(t) to be continuous with α(1)=α(0). Figure 4 shows the principle of the procedure, using a circle as the reference object. In this illustration, the dashed line represents the deformed contour.

The presented method can easily be transferred to the given problem by choosing ϕ(t) and α(t) as random curves. This can be accomplished by choosing randomized curves from the method presented in the previous section, since those curves always fulfill the requirements placed on ϕ(t) and α(t) due to the nature of the FFT. However, this procedure forces the use of discrete variables. For this reason, we define the reference object on a discrete grid using only *N* discrete values. Afterward, the deformation can be calculated as shown in Equation (14) by using discrete functions ϕ(n),α(n)n∈[1,N].

**Figure 5:**
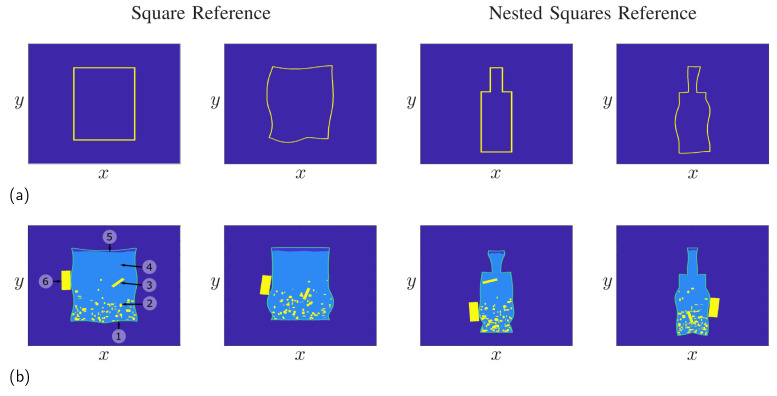
Generation of randomized objects by deformation of a reference object. (**a**) Deformation using a rectangular reference or a reference of nested squares. (**b**) Mine models built from the references. The numbers shown in the mine model on the left side denote the following components: (1) plastic container, (2) additional metal pieces, (3) detonator, (4) explosive material, (5) air gap, and (6) battery.

Figure 5 shows some results for the procedure in the case of a square reference on the left side and a reference consisting of two nested squares on the right side. Here, the parameters α(t) and ϕ(t) were selected as random curves, following the approach in Section 4.1. For instance, for the container, the parameter α(t) was calculated using a standard deviation of σ=3Hz. It is clearly visible that the deformed objects are still closed contours, while the original reference has a strong influence on the final shape of the object. For instance, the number and positions of the edges remain, while straight lines are mapped onto curves. In the following, we will use the two references to build randomized IED models, with some realizations shown in Figure 5b. Here, the mine was modeled as a random plastic container built from an object close to a rectangle for the two mines on the left side. On the other hand, one can build bottle-shaped IEDs from the nested squares reference as shown for the two objects on the right side. In practice, however, IEDs usually have a symmetrical container, as IEDs are usually made from everyday objects, and most available containers are made symmetrically. Therefore, the deformed contours were mirrored to ensure the container of the mine was symmetric. Aside the container, the mine includes the explosive material, a small air gap between the explosive material and the containment, as well as a detonator and a battery, which are used to trigger the explosion. Furthermore, in practice, mines can be filled with additional small pieces of metal to increase the destructive power of the mine when detonated.

The mine models shown were generated completely randomly, as only the battery and the detonator had a constant rectangular shape. However, the placement for the mine was purely random. The metal pieces and the air gap were created using the method in Section 4.1, and the placement of the metal pieces was also assumed to be random, with the density following a gradient as metal pieces typically sink to the bottom of the mine.

## 5. Simulation Procedure

**Figure 6:**
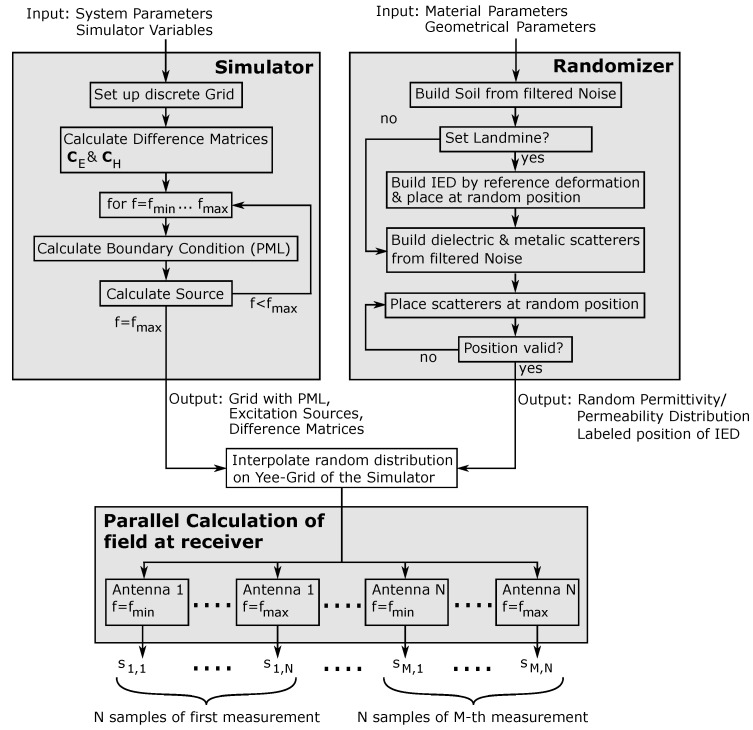
Implementation of the proposed simulation approach.

After introducing the basic principle of the FDFD method in Section 3 and the procedure for creating randomized objects in Section 4, this section aims to give an overview of the implementation steps of the simulator. For this, Figure 6 shows a block diagram of the basic implementation.

The implementation of the simulation framework can be divided into two parts: the one that can be calculated serially on a CPU and the main part, where the equation system is solved in parallel. In this implementation, setting up the simulator and the randomizer can be accomplished independently from each other before merging the outputs of the two blocks. Setting up the empty simulator in the upper left block in Figure 6 requires the system parameters and the simulator variables as the input. Here, the system parameters are the variables describing the radar system itself, namely the operating frequency *f* of the system, which is in the range $f\in [f_{\min},f_{\max}]$, and the spatial position of the radar sensor during each measurement. The simulator variables, on the other hand, are parameters such as the grid spacing of the Yee grid, the parameters required for setting up the boundary conditions, and the overall dimension of the scene. These parameters are used to create the empty discrete Yee grid and calculate the corresponding difference matrices $C_{H}$ and $C_{E}$ from the finite difference approximation introduced in Section 3. Afterward, the TFSF source and the PML are calculated as shown in [35,36] for each discrete frequency point in a for loop. Following this, the outputs of the simulator block are the empty grid with the boundary condition, the excitation source, and the difference matrices. All variables up to this point are independent of the actual material distribution.

In the randomizer block on the upper right side, the material distribution is built on a fine grid. This requires the material parameters as the input, which must be determined by material characterization in advance or can be found in the literature for some selected regions, such as in [38]. Furthermore, the geometrical information for the randomized modeling of targets is required. However, as discussed in Section 4.1, it is not of central importance to model all objects exactly but to present scatterers with a large variance to the classifier. As already mentioned, it is usually a good choice to apply filters with a low standard deviation in the randomizer if no geometric information about the targets is available. To set up the simulation environment, the soil material was calculated with filtered two-dimensional noise as the background material. In the next step, it was decided if the simulated scene contained an IED. If this was the case, then the IED was built as demonstrated in Section 4 and placed at a random position on the grid. Furthermore, if the IED was set, then the position in the simulated environment was stored, since this information is important for the training process. If, on the other hand, the simulated scene did not contain an IED, then the step was skipped, and the scene was labeled accordingly. In the next step, all dielectric and metallic scatterers were calculated and stored separately. Since the scatterers had random sizes and had to be placed at random positions on the grid, the most straightforward approach to achieving this was to place them at random positions and store the coordinates on the grid occupied by the scatterer. Since the scatterers could not overlap in practice, this could be repeated for all scatterers. However, if a collision was detected, then a new random position had to be calculated. As the output, the randomizer block provided a random material distribution of complex permittivities and permeabilities as well as the labeled position of the landmine.

After interpolating the material distribution provided by the randomizer on the empty Yee grid of the simulator, the equation system could be calculated according to Equation (7), and inverting the system for each frequency point led to the electromagnetic field at the receiver. However, since the equation system is independent for each frequency sample and for all positions, the simulation could be parallelized. Therefore, for *M* measurements and *N* samples per measurement, the overall simulation could be divided into M·N independent equation systems. Following this, the simulation framework provided the frequency domain samples $s_{m},n\;,\;m\in \left[1,M\right]\;,\;n\in \left[1,N\right]$ of the received signal, which could be transformed back into the time domain to obtain the range information.

To test the framework, the simulator was implemented for a parallel calculation on an AMD Ryzen Threadripper 2990WX 32 core processor. In the implementation, the simulation of 32 samples could be calculated in parallel. The total run time of the simulation was determined to be 619s, and this time was divided into 19s for the random generation on the grid, 585s for solving the equation systems during the simulation, and 15s for postprocessing. This shows that inversion of the equation system was the most time-consuming step during the simulation. However, the calculation in the frequency domain allows a completely parallel calculation of all frequency samples. Therefore, for practically generating data sets, a calculation on the GPU is advantageous since this allows a massive parallel calculation with which the simulation can be accelerated greatly.

## 6. Randomized Measurement Environment

In the following two sections, we will provide the simulation results of multiple IED realizations. Since the simulations shown here refer to our measurement system, we refer to [19] for a comparison of the obtained simulation results with real measurements of a manufactured test IED in a realistic measurement environment. In reference to [39], we will give a brief overview of the results, since all further simulations are a modification to further adapt the achieved simulation results to the measurement environment.

As stated in the introduction, we assumed a handheld device was used according to [19], which moved with a constant distance of approximately 0.15m above the earth surface. The great advantage of such a system is the possibility to adapt it to environments with strongly developed vegetation, where flying platforms like drones are useless. However, to penetrate the earth surface, low frequencies are required, since increasing the frequency also leads to increased losses, and thus the earth surface becomes non-transparent. Therefore, the frequency range of the proposed system ranged from 0.4GHz up to 4GHz. The number of antenna positions was chosen to satisfy a sampling of the aperture with a spacing of $\frac{\lambda_{\min}}{4}$ according to SAR theory. Here, $\lambda_{\min}$ denotes the minimum wavelength belonging to the highest frequency in the simulated signal. Furthermore, to accelerate the simulation, the number of simulated samples was iteratively increased until the processed images converged. It shows that this was approximately satisfied with a total of N=100 samples. It is known from signal theory that the number of samples has to be chosen in consideration of the highest expected frequency in the signal. For a radar sensor, however, the frequency is directly coupled to the distance from the target. Therefore, increasing the amount of samples also increases the amount of simulation iterations, while the final imaging result remains unaffected as long as the scene is not extended in the range direction. However, it has to be mentioned that increasing the soil permittivity also forces an increase in the number of frequency samples.

In contrast to the measurement system, modeling the measurement environment is a tedious task, since the quantity and form of possible disruptors are basically unlimited. Even if we limited ourselves to only a small set of different interferers, the permittivities could vary strongly. Therefore, we chose to investigate one possible realization of a ground scene with the permittivity values from Table 1.

**Figure 7:**
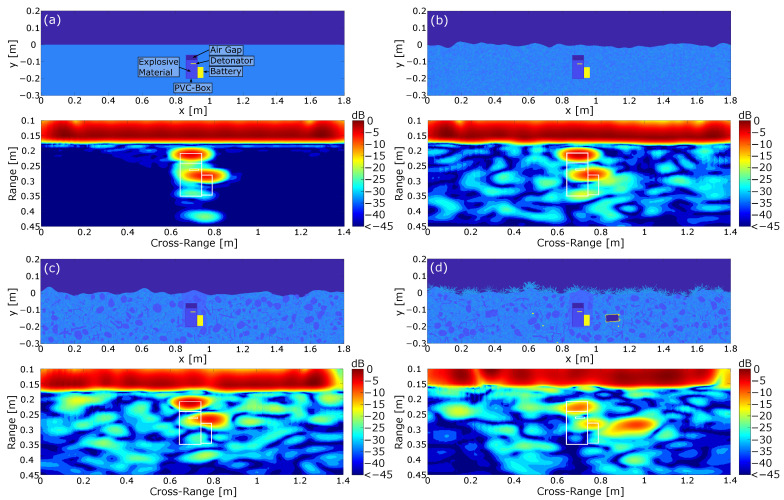
Simulated ground scenes with the corresponding normalized SAR images including the contour of the mine. (**a**) Reference image containing the mine model in a perfectly homogeneous material. All losses were neglected. (**b**) Mine model in an inhomogeneous soil material with a non-planar surface, considering dielectric losses. (**c**) Mine model under the considerations of additional dielectric scatterers inside the ground material. (**d**) Complete scene under the assumption of additional metal interferers and vegetation on the earth surface. [39].

Figure 7 shows four different ground scenes and the related normalized radar images obtained from the simulation after applying a range and cross-range compression on the simulated raw data. Here, the complexity of the scenes increased from a homogeneous lossless ground material with a flat surface in Figure 7a to a more complex measurement environment with numerous additional scatterers and vegetation in Figure 7d. The mine was approximated as a simple rectangular plastic container including the explosive material as well as a metal detonator and an air gap. Furthermore, the mine model included a simplified battery package, which is typically required to trigger the explosion. However, the modeling of more complex mine models is crucial and will be discussed in the further proceedings of this paper.

As can be seen in Figure 7a, the simplified scene with a flat homogeneous ground material resulted in two local concentrated reflections, which can mainly be attributed to the air inclusion and the battery. There was neither a visible concentrated reflection of the detonator nor any disturbing clutter. Removing the detonator and repeating the simulation showed that a small part of the lower reflection associated with the battery was actually caused by the detonator inside the explosive material. However, the resolution of the simulated system was not able to locally resolve the weak reflection for this arrangement. This result can basically be interpreted as the best case scenario, since the flat surface allowed a perfect inversion of the imaging problem while all dielectric losses in the soil material were neglected. In order to better represent reality, the scenario in Figure 7b includes an inhomogeneous lossy material with a non-flat surface. Here, the ground material and the surface were drawn from a random distribution. Comparing the processed image to the previous result in Figure 7a shows that the results were almost similar, since the two reflections were still clearly identifiable. On the downside, the inhomogeneous ground material led to additional clutter, which degraded the image quality. However, the influence of small inclusions in the soil material seems negligible as long as they are small in reference to the wavelength. If we include larger dielectric scatterers like stones and branches in Figure 7c, then this shows that the clutter further increased while the mine reflections were still clearly separable from the background noise. Additionally, Figure 7c includes a slightly different soil material around the mine, which resulted from the digging process. The burial of a landmine typically leads to small air inclusions which decrease the overall relative permittivity. With regard to the processed image, it is shown that the varying permittivity values led to a shift in the battery reflection toward the earth surface. The reason for this can be found in the imaging algorithm, since the varying permittivity values can not be considered in the imaging process.

Finally, the worst case scenario in Figure 7d includes additional metallic scatterers buried in the soil material as well as vegetation on the earth surface. Metallic scatterers in particular present a huge problem in practice, since conventional systems like metal detectors typically have problems identifying mines in the presence of metal pieces. In our case, we implemented a metallic can and various smaller scatterers. Regarding the processed image, it is shown that the strongest reflection was induced by the buried can, while the mine reflection was significantly less pronounced. The small metal scatterers, however, did not lead to strong reflections, which can again be explained by the small diameter in reference to the wavelength. The vegetation on the other side decreased the signal-to-noise ratio inside the soil material significantly since it implied strong losses to the incoming wave.

## 7. Randomized Mine Model

**Figure 8:**
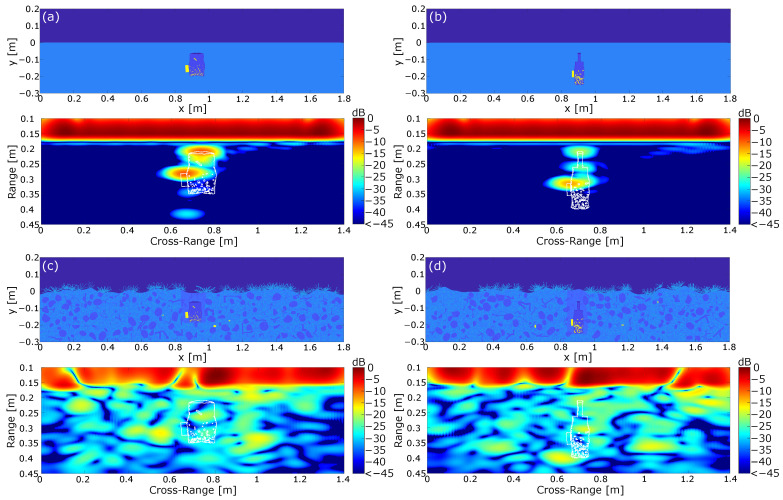
Simulation of randomly generated IEDs in a GPR environment. (**a**) IED build from a square reference in a homogeneous and lossless soil material under the assumption of a flat earth surface. (**b**) IED build from a nested square reference representing a bottle. (**c**,**d**) IEDs in a complex environment containing dielectric and metallic scatterers as well as plants on the earth surface.

Until now, we investigated the influence of a randomized measurement environment. However, as discussed in Section 1, another critical point is the modeling of the IEDs themselves. The reason for this is the unique building type, which drastically complicates the classification process in comparison with professionally built landmines. We already demonstrated previously in Section 4.2 that IEDs can be modeled as continuous deformations of a reference object, with some realizations shown in Figure 5. In this section, we will investigate the behavior of randomized IED models in the simulated measurement environment. This is carried out based on the simplified measurement environment containing a flat earth surface and homogeneous lossless soil material. Subsequently, we evaluate the imaging results in a more complex environment, including dielectric scatterers such as stones and branches, vegetation on the earth surface, and some additional metal scatterers. For the simulation, we limited ourselves to small metallic scatterers, since the simulation of large metallic scatterers in Figure 7d induced a strong distortion of the imaging results. The permittivity values were chosen in reference to Table 1. and the normalized imaging results of the simulation are shown in Figure 8. Here, we used two IED models, where the models in Figure 8a,c are derived from a square reference while the mines in Figure 8b,d were derived from a nested squares reference. Again, the presented IEDs included a metal detonator, as well as a battery and some additional metal pieces. To allow a comparison to the results in Figure 7, the IEDs were placed in the same position, meaning that the top side of the IED was buried at the same distance from the earth surface.

With regard to the mine model in Figure 8a, it is shown that the strongest reflections were still induced by the top side of the mine and the battery. However, compared with the results in Figure 7, it is shown that both reflections were significantly weaker. This can be explained by the fact that the surfaces were not perfectly aligned to the trajectory of the radar system. Therefore, a part of the power was not reflected to the radar system, which finally resulted in a lower amplitude in the range-compressed signal. Another result which can be drawn from Figure 8a is the circumstance that both the detonator and the metal pieces did not lead to significant reflections in the investigated arrangement. This behavior is explainable by the large losses in the explosive material, as the reflected wave was damped to a negligible level before reaching the receiver. Therefore, building an electromagnetic sensing system purely based on detecting metal scatterers is not a promising approach. Furthermore, one could argue that the metal pieces inside the mine are negligible in the given context and can therefore be excluded from the simulation. However, the permittivity values used for the explosive material presented only one possible realization. It was shown in [44] that the losses of commonly used explosive materials vary strongly, with the losses approaching zero for some realizations.

To summarize, it is shown that the reflection pattern of the IED in Figure 8a was close to the reflection pattern of the reference in Figure 7. The main differences were the weaker reflections of the individual components of the IED and the relative shift of the reflections due to the shift of the outer metallic parts. However, the influence of slightly altering the non-metallic geometry of the IED was comparatively small, since it did not significantly influence the reflection pattern for the given IED realization.

The second IED in Figure 8b can be interpreted as a worst case scenario. Here, the mine consisted of a bottle with a height chosen to be 19cm. Furthermore, compared with Figure 8a, the size of the battery was decreased, which resulted in a decreased radar cross-section for both the containment and the battery. Regarding the processed image, it is shown that the reflections of the IED still resulted from the battery and the parallel aligned components on the top of the IED. In comparison with Figure 8a, it turns out that the decreased size of the IED had an effect on the reflection, as all visible reflections were weaker compared with the other investigated IED models, which can be attributed to the smaller radar cross-section. Furthermore, this IED geometry also clearly shows that the internal metal parts led to negligible small reflections. However, when comparing the result to the reference mine architecture in Figure 7 and the IED in Figure 8a, it becomes clear that the reflection pattern changed significantly. The reference mine model and the IED in Figure 8a could both be approximated with two concentrated reflections. The deviating geometry of the second IED model in Figure 8b, on the other hand, showed further deviating reflections, resulting in a new reflection pattern. The additional reflections can be attributed to the fact that the container provided multiple parallel surfaces at different distances, leading to individual reflections of the incoming wave. It can therefore be stated that the geometry of the container is not negligible and must be taken into account during the training process of a classifier. This shows that a classifier for IEDs cannot be trained for a specific mine geometry but must cover many different geometries.

Enhancing the scene with additional dielectric and metallic scatterers as well as vegetation in Figure 8c,d clearly reduced the image quality. In both cases, the mine was not clearly separable from the clutter since the reflection pattern from Figure 8a,b could not be retrieved. Again, the reason can be found in the losses in the plants on the earth surface and the missing compensation of the strongly inhomogeneous soil material. In general, this scene was the closest to the true measurement environment but obviously the hardest to handle in a humanitarian demining scenario.

Overall, the simulations in Section 6 and Section 7 showed good agreement with the previously obtained measurement results with the underlying measurement system presented in [19]. Here, an IED was manufactured from a bottle using home-brew ANFO as the explosive material. Furthermore, the IED contained a battery package on the outside and a detonator inside the explosive material. Carrying out the measurement in a realistic measurement environment and processing the obtained raw data by means of a SAR algorithm showed that the IED could be identified by multiple concentrated reflections that could be assigned to the top of the IED and the outer battery package.

The scenes considered here represent only one possible realization by the simulator. By building up the scene through noise, new scenarios continuously arise, which in turn shows the diverse applicability of the simulation environment.

In summary, the following statements can be derived on the basis of the simulations:

(1) Using a simple mine model in an idealized environment like in Figure 7 leads to a unique radar signature. This signature clearly stood out from the background noise when dielectric interferers were added to the scene.
(2) If the simplified mine is replaced by randomly generated IEDs, then the radar signature is affected. Figure 8a,b shows that the reflection distribution clearly depended on the mine realization. If the transition is made to realistic measurement environments, then a specific signature must still be present in the image, but this can no longer be clearly identified visually.
(3) Since detecting the radar signature of an IED in a realistic measurement environment is a complex task, the use of a trained classifier is of great importance. Even for simplified scenes it was shown that limiting the training data to only one specific type of landmine was not sufficient to cover all types of IEDs.

## 8. Conclusions

In this paper, we presented a statistical simulation concept for the generation of labeled training data using the example of humanitarian demining. The simulator allows generating a realistic scene from a given input of parameters like the soil permittivity and loss tangent, which could also be dispersive since we were applying a frequency domain simulation. Both the system and scene parameters could be chosen arbitrarily, and due to the 2D approximation, rather large extended scenes could be simulated. Furthermore, since the training data for supervised learning applications typically need to be as diverse as possible, two solutions for the generation of random objects were introduced. The methods presented were based on the generation of filtered noise by means of a low-pass filter, exploiting the statistical behavior of white noise in the frequency domain. In addition, more advanced concepts were introduced that could be used to generate random contours through deformation of a reference object, allowing a priori knowledge to be incorporated into the generation process. Subsequently, complex mine models were created based on the presented methods. In the following, more complex scenes were built and simulated by applying a SAR imaging approach including the compensation of the refraction behavior in postprocessing. It was shown by means of simulations that simplified mine models can be reliably detected with the help of GPR systems even under the condition of additional dielectric scatterers. However, further investigations revealed the vulnerability of the GPR principle to highly reflective metal targets. On the other hand, randomized IED models resulted in much weaker reflections, making it harder to distinguish them from background noise. All in all, the presented simulator has been proven to be an effective method for generating diverse training data for supervised learning classifiers, for example in the field of humanitarian demining.

## Figures and Tables

**Table 1:** Permittivity values used for simulation [39].

Subject	$\varepsilon_{r}^{\prime }$	$\varepsilon_{r}^{\prime \prime }$
Vegetation [40]	10	3.8
Branches [41]	5.2	1.5
Stones [42]	5.5	0.2
Soil [43]	9	0.045
Soil around mine	7	0.045
Explosive [44]	4.5	1

## Data Availability

Data are contained within the article.

## References

[1] Shao J., Qu C., Li J., Peng S. (2018). A Lightweight Convolutional Neural Network Based on Visual Attention for SAR Image Target Classification. Sensors.

[2] Palffy A., Dong J., Kooij J.F.P., Gavrila D.M. (2020). CNN Based Road User Detection Using the 3D Radar Cube. IEEE Robot. Autom. Lett..

[3] Ma M., Chen J., Liu W., Yang W. (2018). Ship Classification and Detection Based on CNN Using GF-3 SAR Images. Remote Sens..

[4] Hush Classification with neural networks: A performance analysis. Proceedings of the IEEE 1989 International Conference on Systems Engineering.

[5] Giovanneschi F., Mishra K.V., Gonzalez-Huici M.A., Eldar Y.C., Ender J.H.G. (2019). Dictionary Learning for Adaptive GPR Landmine Classification. IEEE Trans. Geosci. Remote Sens..

[6] Garcia-Fernandez M., Alvarez-Lopez Y., Las Heras F. (2019). Autonomous Airborne 3D SAR Imaging System for Subsurface Sensing: UWB-GPR on Board a UAV for Landmine and IED Detection. Remote Sens..

[7] Šipoš D., Gleich D. (2020). A Lightweight and Low-Power UAV-Borne Ground Penetrating Radar Design for Landmine Detection. Sensors.

[8] Fernández M.G., López Y.Á., Arboleya A.A., Valdés B.G., Vaqueiro Y.R., Andrés F.L.H., García A.P. (2018). Synthetic Aperture Radar Imaging System for Landmine Detection Using a Ground Penetrating Radar on Board a Unmanned Aerial Vehicle. IEEE Access.

[9] García-Fernández M., López Y.Á., Andrés F.L.-H. (2020). Airborne Multi-Channel Ground Penetrating Radar for Improvised Explosive Devices and Landmine Detection. IEEE Access.

[10] Bechtel T., Pochanin G., Truskavetsky S., Dimitri M., Ruban V., Orlenko O., Byndych T., Sherstyuk A., Viatkin K., Crawford F. Terrain Analysis in Eastern Ukraine and the Design of a Robotic Platform Carrying GPR Sensors for Landmine Detection. Proceedings of the 2018 17th International Conference on Ground Penetrating Radar (GPR).

[11] Madavha L., Laseinde T., Daniyan I., Mpofu K. (2020). Functional design and performance evaluation of a metal handheld detector for land mines detection. Procedia CIRP.

[12] Oturak M., Yuksel S.E., Kucuk S. (2023). Multi-source domain adaptation of GPR data for IED detection. SIViP.

[13] Stadler S., Schennen S., Hiller T., Igel J. (2023). Realistic simulation of GPR for landmine and IED detection including antenna models, soil dispersion and heterogeneity. Near Surf. Geophys..

[14] Srimuk P., Boonpoonga A., Kaemarungsi K., Athikulwongse K., Dentri S. (2022). Implementation of and Experimentation with Ground-Penetrating Radar for Real-Time Automatic Detection of Buried Improvised Explosive Devices. Sensors.

[15] Garcia-Fernandez M., Morgenthaler A., Alvarez-Lopez Y., Las Heras F., Rappaport C. (2019). Bistatic Landmine and IED Detection Combining Vehicle and Drone Mounted GPR Sensors. Remote Sens..

[16] Šimić M., Ambruš D., Bilas V. (2023). Landmine Identification From Pulse Induction Metal Detector Data Using Machine Learning. IEEE Sensors Lett..

[17] Bajić M., Potočnik B. (2023). UAV Thermal Imaging for Unexploded Ordnance Detection by Using Deep Learning. Remote Sens..

[18] Lee J., Lee H., Ko S., Ji D., Hyeon J. (2023). Modeling and Implementation of a Joint Airborne Ground Penetrating Radar and Magnetometer System for Landmine Detection. Remote Sens..

[19] Baer C., Schulz C., Just T., Gutierrez S., Orend K., Barowski J., Martinez D., Hattenhorst B., Jebramcik J., Pantoja J. Humanitarian Microwave Detection of Improvised Explosive Devices in Colombia. Proceedings of the 2018 International Conference on Electromagnetics in Advanced Applications (ICEAA).

[20] Gutierrez S., Vega F., González F.A., Baer C., Sachs J. (2019). Application of Polarimetric Features and Support Vector Machines for Classification of Improvised Explosive Devices. IEEE Antennas Wirel. Propag. Lett..

[21] Bestagini P., Lombardi F., Lualdi M., Picetti F., Tubaro S. (2021). Landmine Detection Using Autoencoders on Multipolarization GPR Volumetric Data. IEEE Trans. Geosci. Remote Sens..

[22] Ødegaard N., Knapskog A.O., Cochin C., Louvigne J.-C. Classification of ships using real and simulated data in a convolutional neural network. Proceedings of the 2016 IEEE Radar Conference (RadarConf).

[23] Seyfioglu M.S., Erol B., Gurbuz S.Z., Amin M.G. (2019). DNN Transfer Learning From Diversified Micro-Doppler for Motion Classification. IEEE Trans. Aerosp. Electron. Syst..

[24] Malmgren-Hansen D., Kusk A., Dall J., Nielsen A.A., Engholm R., Skriver H. (2017). Improving SAR Automatic Target Recognition Models With Transfer Learning From Simulated Data. IEEE Geosci. Remote Sens. Lett..

[25] Wang K., Zhang G., Leung H. (2019). SAR Target Recognition Based on Cross-Domain and Cross-Task Transfer Learning. IEEE Access.

[26] Warren C., Giannopoulos A., Gray A., Giannakis I., Patterson A., Wetter L., Hamrah A. (2019). A CUDA-based GPU engine for gprMax: Open source FDTD electromagnetic simulation software. Comput. Phys. Commun..

[27] Giannakis I., Giannopoulos A., Warren C. (2016). A Realistic FDTD Numerical Modeling Framework of Ground Penetrating Radar for Landmine Detection. IEEE J. Sel. Top. Appl. Earth Obs. Remote Sens..

[28] Balsi M., Esposito S., Frezza F., Nocito P., Barone P.M., Lauro S.E., Mattei E., Pettinelli E., Schettini G., Twizere C. GPR measurements and FDTD simulations for landmine detection. Proceedings of the XIII Internarional Conference on Ground Penetrating Radar.

[29] Giannakis I., Giannopoulos A., Davidson N. Realistic Modelling of Ground Penetrating Radar for Landmine Detection Using FDTD. Proceedings of the 15th International Conference on Ground Penetrating Radar.

[30] Pham M.-T., Lefèvre S. Buried Object Detection from B-Scan Ground Penetrating Radar Data Using Faster-RCNN. Proceedings of the IGARSS 2018—2018 IEEE International Geoscience and Remote Sensing Symposium.

[31] Lameri S., Lombardi F., Bestagini P., Lualdi M., Tubaro S. Landmine detection from GPR data using convolutional neural networks. Proceedings of the 2017 25th European Signal Processing Conference (EUSIPCO).

[32] Giannakis I., Giannopoulos A., Warren C., Davidson N. Numerical Modelling and Neural Networks for Landmine Detection Using Ground Penetrating Radar. Proceedings of the 2015 8th International Workshop on Advanced Ground Penetrating Radar (IWAGPR).

[33] Schorlemer J., Schulz C., Pohl N., Rolfes I., Barowski J. (2021). Compensation of Sensor Movements in Short-Range FMCW Synthetic Aperture Radar Algorithms. IEEE Trans. Microw. Theory Tech..

[34] Schorlemer J., Jebramcik J., Rolfes I., Barowski J. Comparison of Short-Range SAR Imaging Algorithms for the Detection of Landmines using Numerical Simulations. Proceedings of the 2021 18th European Radar Conference (EuRAD).

[35] Rumpf R.C., Garcia C.R., Berry E.A., Barton J.H. (2014). Finite-Difference Frequency-Domain Algorithm for Modeling Electromagnetic Scattering From General Anisotropic Objects. Prog. Electromagn. Res. B.

[36] Rumpf R. (2011). Simple implementation of arbitrarily shaped total-field/scattered-field regions in finite-difference frequency-domain. Prog. Electromagn. Res. B.

[37] Yee K. (1966). Numerical Solution of Initial Boundary Value Problems Involving Maxwell’s Equations in Isotropic Media. IEEE Trans. Antennas Propag..

[38] Bechtel T., Truskavetsky S., Pochanin G., Capineri L., Sherstyuk A., Viatkin K., Byndych T., Ruban V., Varyanitza-Roschupkina L., Orlenko O. (2019). Characterization of Electromagnetic Properties of In Situ Soils for the Design of Landmine Detection Sensors: Application in Donbass, Ukraine. Remote Sens..

[39] Schorlemer J., Jebramcik J., Baer C., Rolfes I., Schulz C. A Statistical FDFD Simulator for the Generation of Labeled Training Data Sets in the Context of Humanitarian Demining using GPR. Proceedings of the 2022 IEEE MTT-S International Conference on Numerical Electromagnetic and Multiphysics Modeling and Optimization (NEMO).

[40] Shrestha B.L., Wood H.C., Sokhansanj S. (2007). Modeling of Vegetation Permittivity at Microwave Frequencies. IEEE Trans. Geosci. Remote Sens..

[41] Razafindratsima S., Sbartaï Z.M., Demontoux F. (2017). Permittivity measurement of wood material over a wide range of moisture content. Wood Sci. Technol..

[42] Ulaby F.T., Bengal T.H., Dobson M.C., East J.R., Garvin J.B., Evans D.L. (1990). Microwave Dielectric Properties of Dry Rocks. IEEE Trans. Geosci. Remote Sens..

[43] Curtis J.O. (2001). Moisture Effects on the Dielectric Properties of Soils. IEEE Trans. Geosci. Remote Sens..

[44] Gutierrez S., Just T., Sachs J., Baer C., Vega F. (2018). Field-Deployable System for the Measurement of Complex Permittivity of Improvised Explosives and Lossy Dielectric Materials. IEEE Sens. J..

